# Validation of a New Digital and Automated Color Perception Test

**DOI:** 10.3390/diagnostics14040396

**Published:** 2024-02-11

**Authors:** Alvaro Fanlo-Zarazaga, José Ignacio Echevarría, Juan Pinilla, Adrián Alejandre, Teresa Pérez-Roche, Diego Gutiérrez, Marta Ortín, Victoria Pueyo

**Affiliations:** 1 Ophthalmology Department, Miguel Servet University Hospital, Isabel la Católica, 3, 50009 Zaragoza, Spain; 2 Aragon Institute for Health Research (IIS Aragón), San Juan Bosco 13, 50009 Zaragoza, Spain; 3 DIVE Medical S.L., Paseo Miramón 170, 20014 San Sebastián, Spain; 4 Instituto de Investigación en Ingeniería de Aragón (I3A), Universidad de Zaragoza, María de Luna 1, 50018 Zaragoza, Spain; 5 Department of Microbiology, Pediatrics, Radiology and Public Health, Faculty of Medicine, University of Zaragoza, Domingo Miral, s/n, 50009 Zaragoza, Spain

**Keywords:** color test, color vision deficiency, digital color test, grading color perception, dyschromatopsia, DIVE Color Test, Ishihara, Farnsworth–Munsell

## Abstract

Although color vision deficiencies are very prevalent, there are no ideal methods for assessing color vision in all environments. We compared a new digital and automated method that quantifies color perception for the three protan, deutan, and tritan axes with two of the most commonly used color tests in daily practice: the Ishihara 38 plates test and the Farnsworth–Munsell 100-Hue test. One hundred patients underwent a triple examination composed of the new DIVE Color Test, the Ishihara test, and the Farnsworth–Munsell 100-Hue test. The DIVE Color Test was performed twice in forty participants to assess its repeatability. In the trichromatic group, the mean age stood at 20.57 ± 9.22 years compared with 25.99 ± 15.86 years in the dyschromatic group. The DIVE and Ishihara tests exhibited excellent agreement in identifying participants with color deficiency (Cohen’s kappa = 1.00), while it was 0.81 when comparing DIVE and Farnsworth. The correlation between the global perception values of Farnsworth (TES) and DIVE (GCS) was 0.80. The repeatability of the DIVE Color Test was high according to Bland–Altman analysis with an intraclass correlation coefficient of 0.83. According to Ishihara, the DIVE Color Test proved to be an effective and reproducible tool for red–green color vision deficiency detection, capable of determining the severity of the defect in each of the three axes faster and more accurately than both Ishihara and Farnsworth.

## 1. Introduction

Color vision deficiencies are traditionally classified as congenital or acquired. Around 8% of the male and 0.5% of the female population are born with color vision deficiency; that is, there are 300 million people affected by inherited color vision deficiencies worldwide. In addition, 5 to 15% of the global population could be affected by acquired color deficiencies [1]. The prevalence is mainly related to aging and ocular or visual pathway diseases; therefore, acquired color vision deficiencies are more prevalent in the older population, and their follow-up can be a helpful diagnostic aid [2].

Congenital and acquired color vision deficiencies show different characteristics: the former are usually stable and symmetrical in both eyes, while the latter depend on the stage of the causative disease and may affect both eyes asymmetrically even in different portions of the visual field [1,2,3].

It is widely accepted that color vision testing is essential to having a complete diagnosis and/or monitoring ocular or neurological pathologies that affect millions of people around the globe. Some of these conditions are Parkinson’s disease, multiple sclerosis, retinitis pigmentosa, optic neuritis, Leber hereditary optic neuropathy (LHON), optic neuritis, or Alzheimer’s disease [4,5,6,7,8,9,10,11]. Subclinical losses of color vision have even been found in diabetes mellitus type 2 patients at an early stage of the disease, before other detectable signs of retinopathy [12].

For color vision assessment, most vision standards and practitioners use two analog tests: the Ishihara 38 plates test and the Farnsworth–Munsell 100-Hue test [13,14,15,16]. The Ishihara pseudoisochromatic test is probably the first choice of tests among subjects over 6 years of age for most clinicians, as it is a reliable screening method for establishing the presence of congenital color vision defects. However, it has limitations, as it only assesses color vision perception along the protan and the deutan axes, not the tritan one [17]. Even though, it is not an accurate method to differentiate between red or green color vision defects [18]. Another pseudoisochromatic alternative is the Richmond Hardy–Rand–Rittler test (HRR), which evaluates the three axes and is more effective in grading color defects in mild, moderate, or severe cases [16]. However, neither of these two methods provides a precise quantitative analysis of color discrimination, which is a desirable feature, especially for the better tracking of acquired color vision deficiencies.

On the other hand, the Farnsworth–Munsell 100-Hue rearrangement test (FM 100-Hue) is a widely used method to measure color discrimination ability. Nevertheless, its reliability in detecting the presence or absence of a color vision defect is not particularly strong, as there is no direct correlation between the error score provided by the test and the severity of color vision defects [19]. When implementing this test, it is necessary to know that performance can be enhanced by up to 30% by training. Also, it is time-consuming to perform and to score, especially in more ambiguous results where it may be difficult to determine a confusion axis [20].

In general, analog printed tests such as the above-mentioned present a common weak point, which is the decay along their use. As Plutino et al. concluded, there are clinically significant differences between versions of the same Ishihara test depending on their use, despite their good appearance [21]. In addition, they can be easy to memorize, and they require a specific external illumination that is rarely controlled for during daily clinical practice. Improper illumination affects the correct display of colors and the validity of the tests [22].

The gold standard in color vision testing is the anomaloscope, frequently in the design by Nagel. This test uses Rayleigh’s examination strategy based on color matching and assesses perception along the protan and deutan axes. A posterior version of the anomaloscope, such as the one developed by Oculus, also allows for assessing color perception along the tritan axis by using the Mooreland criterion. Nevertheless, Zabel et al. suggested that the Mooreland test should not be used as a diagnostic test, as it could potentially indicate false deficits in the blue axis [6]. Despite being considered the most reliable method, as it can even classify people with color vision deficits as dyschromats of anomalous trichromats [23], the use of this device is rare in clinical practice since it requires not only a very active comprehension and cooperation by the patient but also must be administered by experienced clinicians [15,24]. In addition, the cost and availability of the anomaloscope can also be a constraint.

Digital color tests aim to avoid some of the disadvantages that analog tests have. For example, the possibility of memorization is canceled by random stimulus displays. Also, external illumination is not required, there is no decay along their use, and they offer the possibility of assessing color perception along larger regions of the color space [25]. Other interesting benefits include easier administration, automated scoring, and integration with electronic health records. However, it should be noted that periodic screen calibrations are required to ensure that the color profile remains correct. Otherwise, as de Fed et al. stated, an incorrect colorimetric characterization of the screen color profile may lead to higher false-positive or false-negative results in dichromatic patients [26]. In addition, they advise the ideal conditions for visual examinations with screens: to perform them in a dark room with the device as perpendicular as possible to the line of sight. Additionally, to prevent alterations in contrast and brightness, they discourage the utilization of external light sources to minimize glare and potential reflections.

Two of the main digital color tests available are the Color Assessment and Diagnosis (CAD) and the Cambridge Color Test (CCT). Despite being a test with proven high accuracy in its full test protocol, CAD administration might be too lengthy for clinical practice, with a duration of around 12–30 min [27,28]. However, it must be said that this test offers a screening test with a duration of around 1.5 min. On the other hand, CCT’s full Ellipse version takes approximately 8 to 15 min to complete, and its trivector screening test lasts around 5 min according to the manufacturer’s specifications.

In the present study, we compare the DIVE Color Test, a new digital, autonomous, and portable color assessment test, to two of the most used and traditionally accepted ones in clinical practice: the Ishihara 38 plates test—an easy-to-understand testing method for all patients, including young children, renowned for its precision in identifying congenital red–green color vision deficiencies [18]—and the Farnsworth–Munsell 100-Hue test, a widely used grading testing method for assessing color discrimination that quantifies global performance by reporting the Total Error Score (TES) value. To achieve this objective, we will compare the accuracy in detecting and classifying color vision deficiencies among the three testing methods. Additionally, we will assess the repeatability of the DIVE Color Test.

## 2. Materials and Methods

In total, 100 participants—65 trichromatics and 35 color-deficient patients—were recruited for the study. The majority of participants were gathered from the pool of patients attending the Ophthalmology Service of the Miguel Servet University Hospital in Zaragoza, Spain. Among them were those with personal concerns about their color vision and others who came for a color vision screening as they had family members affected by some form of color vision defect. Additionally, participants were gathered from the patients’ family members, along with hospital staff through strategically placed posters in high-traffic areas, extending an altruistic invitation to engage in a color vision study. Prospective participants initiated contact with the research team via email and were subsequently arranged for scheduling. When the study was completed, a report with the results of the 3 tests performed was provided to them.

The inclusion criteria were as follows: age between 4 and 60 years [29,30] and written acceptance of the informed consent form by the participants or their parents/legal guardians. Exclusion criteria were as follows: previous ocular, muscular, or orbital surgery; ophthalmological or neuro-ophthalmological disease; poor general health state that did not allow for the clinical protocol; refractive error higher than +/− 3.00 diopters of spherical equivalent; or monocular visual acuity lower than 0.9 in decimal scale (visual acuity was set with the best correction possible).

The study was approved by the local Clinical Research Ethics Committee, Comité de Ética de la Investigación de la Comunidad de Aragón (CEICA), Code PI15/0157. Informed consent was obtained from all participants or their parents/legal guardians before any assessment was carried out, following the Declaration of Helsinki’s determinations.

### 2.1. Equipment

Each patient was tested using three color vision tests: the Ishihara 38 plates test, the Farnsworth–Munsell 100-Hue test, and the DIVE Color Test—DIVE Medical S.L., Spain. The DIVE Color Test is one of the tests that are available on DIVE (Device for an Integral Visual Examination), which is a portable medical device designed to analyze vision as a whole by assessing visual abilities such as oculomotor control, visual acuity, contrast sensitivity, and color perception [31].

Every color test was carried out inside a 12-m-square room without any distracting items. The illumination source was set for the Ishihara test and FM 100-Hue following the manufacturer’s specifications. The table lamp light source specifications were 100–240 V, 50/60 Hz, 120 mA, 10 W, 810 lm, and 6500 K. It provided 900lux, which was assessed using a C.A. 813 luxmeter (Chauvin Arnoux, Paris, France). Color tests were administered binocularly [32], with an undyed optical correction if necessary. All the examinations were carried out by the first author.

#### DIVE Color Test

**Figure 1:**
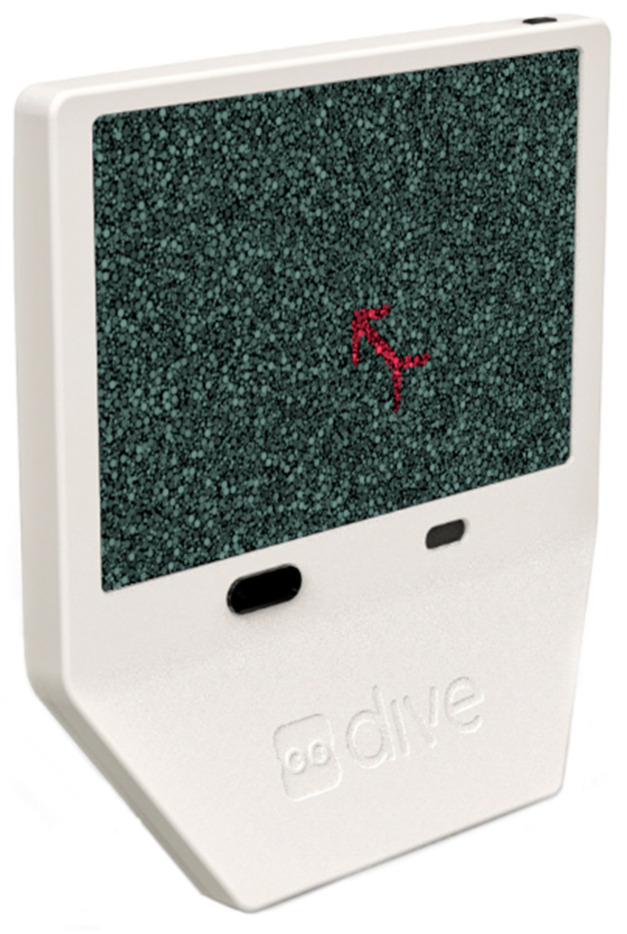
The picture shows the DIVE Color Test set up on a DIVE device.

The DIVE Color Test was set up on a DIVE device with a 12.3-inch tactile screen with a resolution of 2736 × 1824 pixels (see Figure 1). Colors are displayed using an Intel UHD Graphics GPU. The color depth is 8 bits per channel, which provides sufficient granularity to carry out our test without the need to resort to dithering. It was calibrated before each session using an i1Display Pro calibrator manufactured by X-Rite with the following specifications: gamma at 2.20, white dot at 6500 K, and a maximum luminance of 120 cd/m^2^ for the white. We calibrated the screen to the sRGB spec, achievable in mainstream displays these days, enough for a detailed assessment of color vision deficiencies.

**Figure 2:**
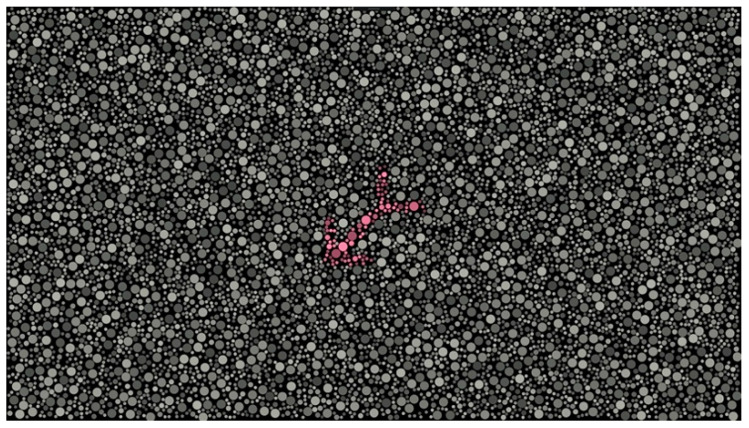
Each digital color test stimulus is composed of an isochromatic background and an arrow. The arrow is shown for a maximum of 3 s; the patient is instructed to touch the head of the arrow on the screen. After the response, a gray screen with a fixation recapture stimulus is shown.

The digital test quantifies color perception thresholds along the three axes using an adaptive method based on patient responses. The test is based on the Stilling [33] and Chibret [34] principles and generates the stimuli automatically. Each stimulus is composed of an isochromatic background and an arrow that measures 4.26° (see Figure 2). The background consists of many circles with different sizes (0.0466° to 0.1244°) and luminance, varying up to 60% from the original luminance value of the color to be displayed. This way, clues based on luminance are masked so that the perception of the stimulus is exclusively reduced to chromaticity differences [35]. The direction of the arrow is chosen randomly among 8 preset options. The stimulus position is obtained by generating a random angle and radius from the center of the screen. The only restriction is that the minimum distance between the center line forming the arrow and the center of the screen must be less than 2°, as the highest density of cones corresponds to the central area of the retina [36].

The patient, seated 50 cm in front of the device, was instructed to touch the head of the arrow on each plate. Each stimulus stayed for a maximum of 3 s. The patient’s response was determined as correct when the patient touched the arrowhead in a delimited area around the target (R = 0.774 cm).

If the patient touched the screen elsewhere or did not touch it anywhere during the time, the answer was determined as incorrect. Between each pair of stimuli, central fixation was encouraged by displaying a gray background with a sounded-rotating star so that potential post-image effects were avoided. During the examination, random control stimuli that could be seen by anyone were automatically displayed to ensure the patient’s understanding of the procedure. This examination could be easily carried out by inexperienced personnel.

The arrow and the background color hues always belonged to the same confusion axes (protan, deutan, or tritan). When the patient touched the screen on the correct spot, the algorithm automatically selected a closer pair of color hues for the arrow and the background. The process continued until the algorithm determined the patient could not distinguish closer color pairs. The color perception threshold for each confusion line was reported using delta E units (dE).

Delta E units quantify the perceptual distance between two color hues (in our case, the arrow from the background) in the three-dimensional L*A*B* color space [37,38]. Delta E units are directly related to human color perception capabilities: for trichromatic eyes, a hue difference from 0 to 1 unit will be invisible. For some trained users, a difference between 1 to 2 units will imply a small difference between both hues. For untrained users, differences in color hues will be slightly noticeable from 2 to 3.5 units [23], being more obvious from 3.5 to 5 units. A value of 6 units is reported to be an obvious difference between hues [39,40].

As those with color vision deficiency have trouble differentiating color hues from the same confusion line, color perception thresholds are expected to be significantly higher than those of normal trichromatic individuals. In addition, a correlation between the severity of color vision deficiency and dE units has been reported [39]. A low-severity defect has a dE of around 22 units, a mild-severity defect has a dE of around 40 units, and a high-severity defect dE is around 60 units [41].

The DIVE Color Test has two methods to assess color perception on the three axes (deutan, protan, and tritan). The first procedure is designed to very accurately grade color perception without prioritizing the duration of the test. The second uses an optimized time–precision psychophysical algorithm that slightly sacrifices accuracy without compromising its clinical reliability, making it suitable for daily clinical practice.

### 2.2. Procedure

Following their recruitment, patients underwent a triple color vision assessment consisting of the Ishihara 38 plates test, the Farnsworth–Munsell 100-Hue test, and the DIVE Color Test. Testing order was randomly determined between patients to mitigate a potential fatigue bias.

A posteriori scoring was needed for both analog tests. The Ishihara test was scored following the specifications of the manufacturer by comparing the responses to the ones suggested in the scoring table. The final color vision defect outcome was noted for a posterior comparison. The FM 100-Hue test responses were introduced in the manufacturer’s Scoring Software, provided by x-rite. The diagnosis of the color vision defect—if there was any—and the general performance value provided by the Total Error Score (TES) were recorded for further analysis.

The repeatability of the DIVE Color Test was assessed in a second testing session with 40 participants (27 trichromats and 13 with color vision deficits) who were selected randomly from the total sample recruited for the study. The second session was performed on the same exploration day after a 30 min break.

**Figure 3:**
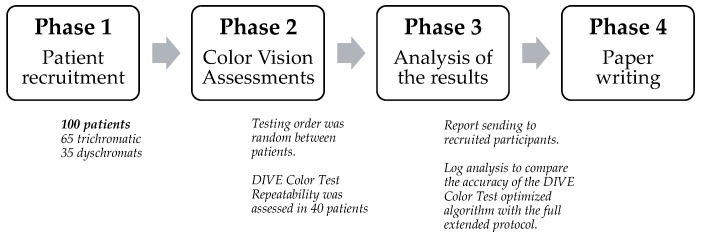
Flowchart of the phases of the protocol followed in the study, with some clarifications added to facilitate comprehension.

Finally, the researchers evaluated the efficacy of using the optimized algorithm to test the same patients. To accomplish this, the technical team analyzed the logs gathered from each patient’s DIVE Color Test assessment and determined the values that would have been obtained if the reduced algorithm had been used. We suggest looking at Figure 3 to facilitate the understanding of the process.

### 2.3. Statistical Analysis

Statistical analyses were performed using the SPSS 24.0 (SPSS Inc., Chicago, IL, USA) statistical software. A *p*-value < 0.05 was considered statistically significant. Descriptive characteristics of the sample were reported as mean and standard deviation (SD), as was the duration of the tests. Comparisons between the study groups were performed with Student’s t-test.

The required sample size for the study was calculated for a sensitivity of at least 90%, a type 1 error of 5%, and a desirable prevalence of dyschromatopsia in our study population of 35%. This estimation provided a minimum of 99 patients to be included in the study. On the other hand, following well-established recommendations to use the sample sizes of other similar articles [42], a total of 100 patients is in agreement with existing evidence [43,44,45].

DIVE Color Test outcomes were reported based on their mean, standard deviation (SD), and range for each axis (protan, deutan, and tritan). According to the one-sample Kolmogorov–Smirnov test, color perception outcomes in the digital test did not follow a normal distribution.

The strength of the agreement between the 3 tests was analyzed using Cohen’s kappa, and its interpretation was performed following Altmann’s recommendations [46].

A metric called the Global Chromatic Score (GCS) was created to compare the results of the DIVE Color Test with the Total Error Score (TES) obtained by FM 100-Hue. The Global Chromatic Score is calculated based on the average color discrimination between the three axes in the DIVE Color Test. Protan dE, deutan dE, and tritan dE denote the color perception thresholds along the protan, deutan, and tritan axes respectively (1).

GCS = (protan dE + deutan dE + tritan dE)/3(1)

Here, both parameters offer a global value of color perception. The correlation between the GCS and the TES from the digital test was determined by a Spearman correlation analysis, as none of these metrics followed a normal distribution.

The repeatability of results in the digital test was determined by a Bland–Altman plot and by the calculation of the intraclass correlation coefficient (ICC) for each axis [47]. Differences between every pair of repeated measurements were evaluated by a paired t-test.

Lastly, the efficacy and viability of the optimized vs. full extended algorithm assessment in the DIVE Color Test were analyzed by comparing both methods’ means, standard deviations, maximums, and minimums, as well as each method’s mean duration.

## 3. Results

### 3.1. Subject Demographics

One hundred subjects were included in the study. The mean age in the trichromatic group was 20.57 ± 9.22 years (range: 4.58–41.72) and 25.99 ± 15.86 years in the color-deficient group (range: 5.08–54.38). In the trichromatic group, 21 participants identified as male (32.3%) and 44 as female (67.7%), while in the color-deficient group, 32 were male (91.4%) and 3 were female (8.6%).

### 3.2. Color Vision Assessment

The DIVE Color Test results mirrored the Ishihara 38 plates test, identifying 65 individuals as normal trichromats and 35 as color-vision-deficient. Both showed perfect agreement, boasting a Cohen’s kappa of 1.0. In contrast, the Farnsworth–Munsell 100-Hue test identified 73 participants as trichromatic and 27 as having color vision deficiencies, so the agreement slightly decreased to 0.814 when comparing DIVE with FM 100-Hue.

Among those with color vision deficiencies, the DIVE Color Test identified 12 individuals with a protan color vision deficiency and 23 with a deutan deficiency. In contrast, the Ishihara 38 plates test detected 2 individuals as protan and 33 as deutan. The Farnsworth–Munsell 100-Hue test categorized 9 as protan and 7 as deutan, but for 11 individuals, this test could not determine the specific type of color vision defect (refer to Table 1). When considering all three tests, a moderate agreement emerged in identifying the specific color defect. The agreement between DIVE and Ishihara was 0.720 and 0.613 between DIVE and FM 100-Hue.

Additionally, we compared the color-perception-grading ability of the DIVE Color Test against FM 100-Hue, obtaining a correlation of 0.80 (*p* < 0.01) between the respective GCS and TES values.

Mean color vision outcomes from the DIVE Color Test and FM 100-Hue are collected in Table 2. According to the mean results provided by the DIVE Color Test, significant disparities between the trichromatic and color vision-deficient patients were found. These differences are statistically significant for color perception along the deutan and protan axes among the three study groups (trichromatic, protan, and deutan). Logically, no differences were observed between the groups regarding color perception on the tritan axis, as no patients with a color vision defect on this axis were recruited.

### 3.3. Repeatability of the DIVE Color Test

**Figure 4:**
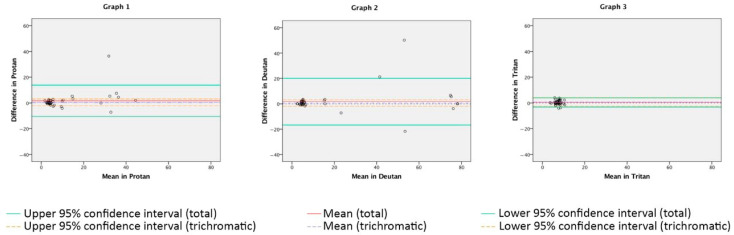
Graph 1 shows the Bland–Altman plot of agreement between the first and second examinations in the DIVE Color Test for the protan axis. Graph 2 and Graph 3 show the same information for the deutan and tritan axes, respectively. Agreement between both sessions is high for the three axes. The mean difference between the trichromatic and color-vision-deficient participants is notable for the protan and deutan axes. The trichromatic group gathers data near the 0 difference between both sessions, together with a low dE; meanwhile, the color-vision-deficient group gathers data near the 0 difference as well, but the means are substantially higher than the trichromatic group since their dEs are significantly higher. For the tritan axis, all data are close to 0 difference between both sessions, and the mean dE results are low since there were no tritan patients diagnosed in the color-vision-deficient sample.

The repeatability between the two sessions was measured by a Bland–Altman plot for each axis (see Figure 4). The mean intraclass correlation coefficient for both sessions for the deutan, protan, and tritan axes was 0.83, showing strong reliability according to Landis and Koch [48].

The results of the DIVE Color Test showed no differences in diagnosis between the first session and the second session. The mean differences between both sessions were as follows: 1.62 for the protan axis (*p* = 0.107), 1.86 for the deutan axis (*p* = 0.221), and 0.38 for the tritan axis (*p* = 0.189). The results of each session were also analyzed differentiating between trichromats and color-deficient patients (Table 3). The results for both groups were very consistent between sessions. For the trichromatic group, differences were always lower than 1.00 dE. For the color-vision-deficient group, differences were around 4.00 dE. Those perceptual differences are almost unnoticeable for each population.

The mean exploration time for the full extended protocol in the digital test for the trichromatic group was 7.41 min (SD: 1.75) and 8.63 min (SD: 1.94) for the color-deficient group (Table 4). The mean Farnsworth–Munsell 100-Hue administration time for the trichromatic group was 11.41 min (SD: 2.59) and 17.11 min (SD: 2.75) for the color deficiency group. The mean Ishihara 38 plates test exploration time was 2.23 min (SD: 0.17) for the trichromatic group and 2.52 min (SD: 0.32) for the color-deficient group.

A significant reduction in the examination time was achieved by applying the optimized algorithm in the DIVE Color Test. For the trichromatic participants, the mean exploration time was reduced to 1.07 min (SD: 0.34) from 7.41 in the extended protocol. For the dyschromatic participants, the evaluation time was reduced to 1.34 min (SD: 0.36) from 8.63 in the extended protocol. After applying this optimized algorithm, the diagnosis changed in only one patient, from trichromatic to having a tritan defect.

## 4. Discussion

Suffering from color vision impairment directly affects many aspects of daily life. This condition can influence education, occupation performance, social and emotional relationships, personal care, and access to entertainment and information, as Stoianov et al. showed [49]. Given this fact, it is important to diagnose color deficiencies during childhood so that condition awareness, school materials, user interfaces in devices, and future professional orientation are adapted from the first years of life.

The most common types of color deficiencies are congenital, meaning that they are lifelong and incurable. Color vision mainly develops during the first 4 years of life [50], and there is a gradual and global decline in color vision as people age [51], with a more significant impact on the blue–yellow spectrum [52].

Practitioners agree that a color vision assessment is useful to have a complete diagnosis and/or to monitor some prevalent ocular or neurological pathologies that debut with acquired color perception deficiencies, such as retinal detachment, vascular and hematologic diseases, glaucoma, diabetic retinopathy, hypertension, progressive cone dystrophies, hereditary optic atrophy, optic nerve diseases, or the effect of toxic agents such as alcohol or tobacco, as well as neurological disorders like Alzheimer’s disease [4,5,6,7,8,9,10,11,12,53]. Nevertheless, despite this significant need, color vision assessment is not a routinely performed examination in ophthalmology practice. The reasons underlying this absence in routine clinical practice might be a lack of fast and accurate tests able to fully assess color discrimination in most patients. Additionally, these tests should be independent of lighting conditions, self-correcting, and easily integrable with electronic medical records.

As mentioned above, both the Ishihara and FM 100-Hue tests were chosen because of their strengths and relevance in clinical practice and their popularity, despite not being the most adequate in classifying chromatic defects [18,54,55]. The Ishihara 38 plates test was also chosen as the main color vision screening test since it is very reliable in detecting red–green color vision defects [18] and is easy to understand and fast to perform for every patient. The Farnsworth–Munsell 100-Hue test was chosen as it is a widely used grading method for assessing color discrimination. The direct comparison between the DIVE Color Test and the Farnsworth–Munsell 100-Hue test, relative to overall color vision performance, was conducted by analyzing their respective outcomes. This evaluation included the GCS from the DIVE Color Test and the TES from the FM 100-Hue test.

Regarding the comparative assessments between the three tests, the DIVE Color Test emerges as a favorable choice because of its demonstrable repeatability and high accuracy. It effectively identifies individuals with color vision deficiencies, closely matching the Ishihara test’s skill in detecting red–green color vision deficiencies. In comparison with the FM 100-Hue test, the DIVE Color Test proves to be able to grade the color perception of patients in an efficient but faster way. Notably, it offers the added benefit of classifying and grading the severity of color vision defects without requiring subsequent corrections. This is crucial considering potential errors from manual corrections in tests like FM 100-Hue or Ishihara. Even with FM 100-Hue’s software version, subjective defect classification might cause clinician discrepancies. Additionally, its consistent illumination control minimizes errors related to incorrect lighting in analog tests. This, combined with its portability, easy-to-use design needing minimal training, integration into electronic medical records, and instant post-test result display, stands out as a significant strength.

For this particular research study, administration time was not a priority, and the research team executed a psychophysical algorithm with a high-precision setting to evaluate color perception along each line as accurately as possible. However, the digital test reached a mean time-saving value of around 35% per trichromatic patient and around 50% per color-deficient one compared to the mean administration time for the FM 100-Hue test [20,56]. As a reflection, the extended assessment protocol used in the DIVE Color Test could potentially be useful for monitoring slight changes in color perception caused by certain pathologies.

On the other hand, the effectiveness and feasibility of an optimized algorithm was evaluated. This refinement enabled the examination time to be reduced to approximately one minute, maintaining near-perfect accuracy in evaluations. This testing approach facilitates the integration of the DIVE Color Test into more accessible environments, such as standard ophthalmology practices.

In future work concerning disparities in the classification of color vision defects, it would be an interesting approach to compare the DIVE Color Test’s diagnosing accuracy with other tests whose validities in this aspect are more contrasted, such as the Anomaloscope test, the Color Assessment and Diagnosis test, or the Cambridge Colour Test. However, the DIVE Color Test classification of color vision defects based on the axis with the poorest color perception outcomes seems to be more precise, consistent, and accurate than the other two analog testing methods.

The DIVE Color Test—in its Eye Tracking version—has also been used to analyze how color vision evolves from infancy to adolescence. By using this test method, normative curves were generated after recruiting 1498 healthy children aged 2 to 15 years. In addition, this study further investigated the influence of prematurity on color vision. [50].

## 5. Conclusions

In a comparison between the three tests, the DIVE Color Test is positioned as a competitive alternative to the Ishihara 38 plates test in the detection of color vision defects and the Farnsworth–Munsell 100-Hue test in the grading of color perception. Without compromising its duration, it offers the advantages of a digital self-correcting test that is capable of assessing defects along the three axes with accuracy, making it an attractive and viable solution for daily clinical practice.

## Figures and Tables

**Table 1:** Classification for each color test used in the present study: the Ishihara 38 plates test; our digital test, the DIVE Color Test; and the Farnsworth–Munsell 100-Hue test.

	Trichromatics	Color-Deficient	Protan	Deutan	Ambiguous
Ishihara 38 plates	65	35	02/35	33/35	0/35
	(65.0%)	(35.0%)	(5.7%)	(94.3%)	(0.0%)
DIVE Color Test	65	35	12/35	23/35	0/35
	(65.0%)	(35.0%)	(34.3%)	(65.7%)	(0.0%)
FM 100-Hue	73	27	09/27	07/27	11/27
	(73.0%)	(27.0%)	(33.0%)	(27.0%)	(40.0%)

Note: Both the Ishihara and DIVE Color Test set the same 35 patients with color deficiency from the 100 participants while Farnsworth–Munsell 100-Hue set 27. DIVE Color Test classified the patients with color deficiency as 12 protan, 23 deutan, and 0 ambiguous, and they were able to classify every color vision defect. Ishihara 38 plates only classified 2 participants as protan and the rest (33) as deutan. Farnsworth–Munsell 100-Hue classified 9 dyschromats as protan and 7 as deutan. For 11 participants, this test was unable to determine their color vision defect.

**Table 2:** Mean color perception outcomes in DIVE Color Test and Farnsworth–Munsell 100-Hue.

DIVE Color Test Outcomes								
Diagnosis		Trichromatic		Protan-Deficient		Deutan-Deficient		*p*
		Mean (Min–Max)	Standard Deviation	Mean (Min–Max)	Standard Deviation	Mean (Min–Max)	Standard Deviation	
Confusion lines	Protan	4.44 dE (2.54–10.63)	1.53	41.78 dE (22.48–66.75)	14.06	14.38 dE (4.38–66.75)	12.23	<0.001
	Deutan	5.38 dE (2.51–9.40)	1.46	17.69 dE (11.20–24.61)	4.44	70.99 dE (27.97–78.05)	14.35	<0.001
	Tritan	8.45 dE (3.30–13.04)	2.36	8.20 dE (5.02–14.59)	2.70	12.00 dE (5.28–75.92)	14.05	0.177
GCS		6.09 (3.24–10.52)	1.47	22.55 (13.52–33.15)	6.53	32.46 (17.52–55.70)	8.83	<0.001
Farnsworth Munsell 100-Hue outcomes								
Total Error Score		18.00 (0.00–72.00)	19.18	147.00 (64.00–147.00)	76.33	217.04 (72.00–640.00)	149.61	<0.005

Note: Outcomes in both color tests for the trichromatic and color-deficient (protan and deutan) participants are on each confusion line. Outcomes for typical trichromatics are highly consistent on each axis according to the range and the standard deviation. As we expected, mean results in the protan line for the protan participants are significantly higher than those on the deutan and tritan axis. The same phenomenon occurs for the deutan group; results on the deutan confusion line are significantly higher than on the other lines. Nevertheless, both protan and deutan do not only have their axes altered. Protans have higher results on the deutan line than trichromats, and deutans have higher results on the protan line than trichromats. Values for the tritan axis remain constant between groups since there were no tritans in the sample.

**Table 3:** Comparison of the mean results in the first session and the second session for the trichromatic and the color-deficient group.

DIVE Color Test Repeatability										
		First Session dE (SD)	Second Session dE (SD)	Mean Difference dE			First Session dE (SD)	Second Session dE (SD)	Mean Difference dE	Paired Samples Test (*p*)
Trichromatic group	Protan	4.16 dE (SD: 1.12)	3.74 dE (SD: 1.34)	0.42 dE	Color-deficient group	Protan	25.96 dE (SD: 14.88)	21.84 dE (SD: 12.28)	4.12 dE	0.107
	Deutan	5.07 dE (SD: 1.26)	4.42 dE (SD: 1.14)	0.65 dE		Deutan	54.37 dE (SD: 28.05)	49.97 dE (SD: 28.15)	4.40 dE	0.221
	Tritan	7.56 dE (SD: 1.74)	6.91 dE (SD: 1.60)	0.65 dE		Tritan	7.39 dE (SD: 1.62)	7.67 dE (SD: 1.93)	0.28 dE	0.189

Note: Results for the trichromatic group are very consistent and are not clinically significant as the differences between the first session and the second session are always lower than 1.00 dE. For the color-deficient group, differences between both sessions are higher but also of low clinical significance for this population as those color differences are almost unnoticeable for them.

**Table 4:** Comparison of the mean results in the first session and the second session for the trichromatic and the color-deficient groups.

DIVE Color Test Protocol			Full Extended		Optimized	
			Mean (Min–Max)	Standard Deviation	Mean (Min–Max)	Standard Deviation
Trichromatics	Confusion lines	Protan	4.44 dE (2.54–10.63)	1.53	7.22 dE (4.09–14.84)	2.22
		Deutan	5.38 dE (2.51–9.40)	1.46	8.09 dE (7.90–10.79)	0.74
		Tritan	8.45 dE (3.30–13.04)	2.36	13.26 dE (7.87–67.98)	15.34
	GCS		6.09 (3.24–10.52)	1.47	9.53 (6.63–27.58)	5.14
	Test duration		7.41 min (2.70–11.77)	1.75	1.07 min (0.67–1.84)	0.34
Dyschromats	Confusion lines	Protan	36.88 dE (7.78–66.75)	23.35	37.00 dE (1.35–62.43)	22.74
		Deutan	38.17 dE (12.79–78.05)	24.55	37.85 dE (10.79–73.18)	23.35
		Tritan	8.45 dE (5.28–15.25)	3.24	9.21 dE (4.57–17.31)	3.26
	GCS		27.83 (13.52–55.70)	7.00	28.02 (15.36–38.92)	6.84
	Test duration		8.63 min (2.61–10.76)	2.07	1.34 min (0.76–2.03)	0.36

Note: The optimized method’s time reduction compared with the full extended protocol holds immense clinical significance and could be pivotal in integrating this test into daily clinical practice. Note that the mean differences between both methods are not clinically relevant, except in the case of the measurements on the tritan axis in trichromatic patients. This may be due to the results obtained from a specific patient who can be considered an outlier, which caused the standard deviation to increase as well.

## Data Availability

The data supporting the conclusions is not accessible due to commercial purposes.
